# Radiosynthesis and preclinical evaluation of [^11^C]Methyl-V-0219, a small molecule glucagon-like peptide-1 allosteric modulator for PET brain imaging

**DOI:** 10.1186/s41181-026-00486-4

**Published:** 2026-08-14

**Authors:** Francesco Lechi, Amina Khalil, Pablo Novales Bautista, Jakob Öberg, Lorenzo Jacopo Ilic Balestri, Olof Eriksson, Jonas Eriksson, Luke R. Odell

**Affiliations:** 1 Science for Life Laboratory, Department of Medicinal Chemistry, Uppsala University, Dag Hammarskjölds väg 14C, 3tr, 751 83 Uppsala, Sweden; 2 Department of Medicinal Chemistry, Uppsala University, Uppsala, Sweden; 3 PET Center, Uppsala University Hospital, Uppsala, Sweden

**Keywords:** Carbon-11, Radiochemistry, GLP-1R, PET imaging, Small molecule, Suzuki coupling

## Abstract

**Background:**

Glucagon-like peptide-1 receptor (GLP-1R) is a clinically validated therapeutic target for the treatment of obesity and type 2 diabetes. GLP-1Rs expressed in the central nervous system (CNS) regulate appetite and are therefore particularly important in the context of weight loss. There is thus an emerging need for efficient and brain-penetrating Positron Emission Tomography (PET) technologies to study the distribution of GLP-1R in the CNS and facilitate the development of novel GLP-1R-targeted therapeutics. However, currently established GLP-1R PET tracers are peptide-based and exhibit limited blood–brain barrier (BBB) penetration, restricting their use for imaging central GLP-1R expression. V-0219, a small molecule GLP-1R positive allosteric modulator, represents a potential scaffold for the development of BBB-penetrating PET tracers targeting incretin receptor systems in the brain.

**Results:**

Here, we report the radiosynthesis and preclinical evaluation of [^11^C]Methyl-V-0219, a carbon-11 labelled analogue of V-0219. [^11^C]Methyl-V-0219 was synthesized using a Pd(0)-mediated Suzuki–Miyaura coupling reaction and obtained with a radiochemical yield of 36 ± 18% (n = 16) and radiochemical purity of 98.6 ± 1.6% (n = 16). In vitro autoradiography demonstrated retained binding to GLP-1R–positive tissues, although binding to glucose-dependent insulinotropic polypeptide (GIP) and glucagon (GCG) receptors was also observed.

In vivo PET imaging was performed in rats and pigs and compared with the well-established GLP-1R tracer [^68^Ga]Ga-DO3A-Exendin-4. Dynamic PET imaging demonstrated rapid brain uptake of [^11^C]Methyl-V-0219 followed by progressive washout, indicating BBB penetration but limited retention in the brain. In contrast to [^68^Ga]Ga-DO3A-Exendin-4, [^11^C]Methyl-V-0219 did not demonstrate detectable retention in GLP-1R–rich tissues, including the pituitary gland and pancreas. Instead, prominent uptake was observed in the liver, intestines, and glandular tissues, consistent with hepatobiliary clearance and nonspecific accumulation of a lipophilic compound. Furthermore, blocking and competition studies did not alter tracer brain kinetics, suggesting no detectable displaceable binding in vivo.

**Conclusion:**

Taken together, these findings demonstrate the feasibility of developing small–molecule–based PET tracers targeting incretin receptor systems, while highlighting the challenges associated with achieving sufficient in vivo specificity for brain GLP-1R imaging.

**Supplementary Information:**

The online version contains supplementary material available at 10.1186/s41181-026-00486-4.

## Background

The growing prevalence of metabolic disorders, including type 2 diabetes (T2D) and obesity, represents a major global health challenge, contributing substantially to morbidity and mortality while placing an increasing burden on healthcare systems worldwide (WHO Global Burden of Disease Collaborative Network 2021)). Consequently, there is a pressing need to improve our understanding of the underlying disease mechanisms and to develop more effective therapeutic strategies (WHO Global Burden of Disease Collaborative Network^ 2021^). In the pursuit of effective treatments and a better understanding of disease mechanisms, numerous biological targets have been explored. Among these, the glucagon-like peptide-1 receptor (GLP-1R) has emerged as a clinically validated therapeutic target, with several approved drugs demonstrating substantial benefits for the treatment of T2D and obesity (Drucker 2006). Mono- and dual receptor targeting incretin mimetics, such as semaglutide (GLP-1R mono-agonist) and tirzepatide (GLP-1R/glucose-dependent insulinotropic polypeptide (GIP) receptor dual agonist), have recently been approved for treatment of T2D and obesity (Sinha et al. 2023). Incretin mimetics have demonstrated significant benefits not only in glucose control but also in weight loss, which has heightened the interest in understanding the role of the GLP-1R, particularly in the brain and specifically the areas involved in satiety and appetite control (Drucker 2006). Given the involvement of GLP-1R in metabolic regulation, glucose homeostasis, appetite control, and neuroprotection, specific imaging agents are needed to visualize GLP-1R distribution, receptor availability, and drug occupancy in vivo by Positron Emission Tomography (PET) (Sinha et al. 2023). However, an imaging agent capable of passing the Blood Brain Barrier (BBB) and accurately mapping brain GLP-1R distribution is currently lacking. Established PET agents based on the exendin-4 peptide have demonstrated excellent in vitro and in vivo properties for imaging and quantification of peripheral and pancreatic GLP-1R (Selvaraju et al. 2013); (Eriksson et al. 2022)). However, exendin-4-based tracers are relatively large (≈4 kDa) and hydrophilic, limiting blood–brain barrier permeability and rendering them suboptimal for visualizing central GLP-1R (Deden et al. 2021). Currently, only Circumventricular Organs (CVOs) and brain regions lacking an intact BBB can be readily probed with exendin-4 PET ligands (Khalil et al. 2025). Novel non-peptidic PET radioligands for central GLP-1R imaging is therefore needed and could provide critical insights into receptor dynamics in the brain, particularly therapeutic effects and disease mechanisms related to appetite control and satiety.

**Fig. 1:**
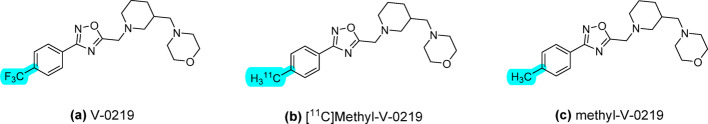
Chemical structures of V-0219 and related compounds. The compounds of interest in this study: **A** V-0219, a GLP-1R positive allosteric modulator and proposed “diabesity” treatment drug; **B** [^11^C]Methyl-V-0219, a proposed PET tracer targeting GLP-1R; **C** reference compound for development of quality control and purification methods. Structural differences are highlighted in blue

The molecule V-0219 (Fig. 1A) is a GLP-1R positive allosteric modulator first reported in 2022 (Decara et al. 2022); (Bueno et al. 2020a); (Méndez et al. 2020). Unlike orthosteric ligands that bind to the extracellular peptide-binding site, positive allosteric modulators interact with a distinct allosteric site (Jeffrey Conn et al. 2009)). Although the precise binding site of V-0219 has not been experimentally resolved, small-molecule GLP-1R allosteric modulators are thought to bind within allosteric pockets in the receptor transmembrane domain (Bueno et al. 2020b). Given its positive effects on insulin secretion and on reducing food intake in mice, V-0219 has been proposed as a potential therapy for diabetes and obesity. As a small, non-peptidic molecule with physiochemical properties more compatible with BBB penetration than exendin-based peptides, V-0219 was considered a potential scaffold for a first-in-class tracer targeting GLP-1R in the brain. In this study, a carbon-11-labeled analogue of V-0219 was developed to enable its biological evaluation as a BBB-penetrating PET tracer.

The Suzuki–Miyaura reaction is a well-established palladium-catalyzed C–C coupling strategy with broad applications in medicinal chemistry and pharmaceutical synthesis (Farhang et al. 2022). In carbon-11 radiochemistry, Suzuki–Miyaura C-methylation provides a complementary strategy to conventional heteroatom ^11^C-methylation, enabling direct incorporation of carbon-11 into carbon frameworks. This approach is particularly useful when the desired radiolabel is part of an aryl, alkenyl, or related carbon skeleton rather than attached to a heteroatom. In recent years, this strategy has been applied to the synthesis of several carbon-11 PET tracers including [^11^C]ATRA, [^11^C]cetrozole, [^11^C]CIMBI-712, [^11^C]dehydropravastatin, 17-[^11^C]HAD, [^11^C]MENET, [^11^C]M-MTEB, N-(4-[^11^C] ethylphenyl)propionamide, [^11^C]palmitate, [^11^C]PSPA-4, and [^11^C]toluene derivatives ( (Rokka et al. 2021); (Milicevic Sephton et al. 2020); (Koyama et al. 2013); (Doi et al. 2009); (Watanabe et al. 2014). Its value in carbon-11 chemistry lies in efficient C–C bond formation, access to carbon-labeled analogues that are not readily obtained by heteroatom methylation, and compatibility with the short half-life of carbon-11 (20.4 min).

For these reasons, Suzuki–Miyaura ^11^C-methylation was selected as the strategy to radiolabel a methyl analogue of V-0219 (Fig. 1B, C), (Farhang et al. 2022; Jonasson et al. 2020; Watanabe et al. 2014; Hostetler et al. 2005; Rokka et al. 2019). Importantly, there were no significant differences in functional activity between the methyl (CH_3_-V-0219) and trifluoromethyl (V-0219) analogues, as replacement of the trifluoromethyl group with a methyl substituent did not improve GLP-1 potentiation in the reported cAMP assay (Decara et al. 2022). This study focuses on two main objectives: establishing and optimizing a reaction protocol for the synthesis of [^11^C]Methyl-V-0219, and evaluating the resulting compound in vitro and in vivo as a brain-penetrant GLP-1R PET tracer.

## Material and methods

All reagents and solvents for synthesis and quality control (QC) were purchased from Merck (Germany) or Thermo Fisher Scientific (Massachusetts, USA). Reformulation was performed using Sep-Pak Plus light C18 cartridges (Waters, Massachusetts, USA). The custom-built Tracer Production System (TPS) module was used to perform the process from the synthesis of the labeled compound to the formulation of the final product ready for injection. HPLC purification was performed on an Agilent 1260 Infinity II system (Agilent Technologies, California, USA) paired with a Gemini C18 column (250 × 10 mm, 5 µm, 110 Å; Phenomenex, CA, USA). A radio-HPLC Agilent 1290 Infinity II system (Agilent Technologies, California, USA) paired with a Chromolith Performance RP-18e (100 × 4.6 mm, Merck, Germany) was used to determine radiochemical purity (RCP).

For a detailed precursor synthesis and reference chemistry, quality control, and HPLC separation methods, please see the Supplementary Methods and Supplementary Tables S1 and S2.

The synthesis and characterization of the precursor compounds used for radiolabeling are described in the Supplementary Methods. Representative NMR and mass spectra are provided in Supplementary Figures S6–S8.

### [^11^C]Methyl iodide production

Radiolabeling of [^11^C]-methyl-V0219 was performed using a custom-built TPS module. [^11^C]CO_2_ was cyclotron produced (45 μA current, 17 MeV, Scanditronix MC-17) via the ^14^N(p,α)^11^C reaction by protonic bombardment of nitrogen gas (AGA, Nitrogen 6.0) containing 0.05% oxygen. [^11^C]CO_2_ was transferred to the synthesis module and trapped in LiAlH_4_ (300 µL, 0.2 M) in tetrahydrofuran (THF). After evaporation of the THF, aqueous HI (600 µL, 57%) was added and the mixture was heated (1 min, 135 °C), to generate [^11^C]CH_3_I which was transferred in a stream of nitrogen gas to the Suzuki–Miyaura coupling reaction vessel.

### Synthesis of [^11^C]Methyl-V-0219

**Scheme 1:**
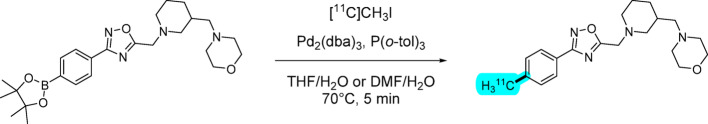
Synthesis of [^11^C]Methyl-V-0219

The radiolabelling of [^11^C]Methyl-V-0219 was performed using a palladium-catalyzed Suzuki–Miyaura coupling reaction (Scheme 1). Pd_2_(dba)_3_ (0.9–1.2 mg, 1.0–1.3 µmol) and P(*o*-tol)_3_ (1.2–1.5 mg, 3.9–4.9 µmol) were dissolved in 1050 µL THF or DMF in a 1.5 mL flat-bottom vial to prepare a stock solution. The solution was degassed with nitrogen, 100 ml/min for 1 min. An aliquot of 350 µL of this solution corresponding to approximately 0.33–0.43 µmol Pd_2_(dba)_3_ and 1.3–1.6 µmol P(o-tol)_3_, was added to a 1 mL vial containing K_2_CO_3_ (0.4–1.2 mg, 2.9–8.7 µmol) and pinacol boronic ester precursor **1d** (1–1.2 mg, 2.1–2.5 µmol) (Supplementary Scheme 1). MilliQ water (40 µL) was added, and after additional degassing (N_2_, 100 ml/min, 1 min) the vial was transferred to the TPS module and [^11^C]CH_3_I was introduced, and the reaction mixture was heated at 70 °C for 5 min.

### Purification and formulation

[^11^C]Methyl-V-0219 was purified by preparative HPLC, injecting the reaction mixture onto a C18 column and employing a gradient mobile phase consisting of ammonium formate (AMF, 50 mM, pH 3.50) and acetonitrile (ACN, ≥ 99.9%, HPLC grade) (Method: 30–70% ACN over 15 min) (t_R_ ≈ 7.5 min). The product-containing fraction was collected and [^11^C]Methyl-V-0219 was trapped on a C18 SPE cartridge, washed with sterile water (5 mL), and then eluted in ethanol (absolute, 99.9%) followed by phosphate-buffered saline (PBS, 7 mL). The resulting formulation contained [^11^C]Methyl-V-0219 in a 5–6 mL solution of PBS with approximately 10% (v/v) ethanol. RCP was assessed via radio-HPLC using a monolithic C18 column with a gradient AMF/ACN mobile phase (Method: 10–40% ACN over 10 min) (t_R_ = 5 min) (Supplementary Figure S1).

### Quantification of [^11^C]Methyl-V-0219

The concentration of [^11^C]Methyl-V-0219 in the final formulation was determined from the integrated UV peak area using an external calibration curve. The UV calibration curve was generated from duplicate HPLC injections at five concentrations of methyl-V-0219 in ACN (0.4, 0.8, 1.2, 1.6, 2.0 µg/mL) (HPLC method: gradient AMF/can mobile phase, 10–40% ACN over 10 min). The integrated area peak values were plotted on a linear regression curve using GraphPad Prism 10.4 (GraphPad Software Inc., USA) (Supplementary Figure S2). The decay-corrected molar activity (A_m_) in the final solution was calculated according to the Equation below.

$$ A_{m} = \frac{{Activity_{{A^{*} }} \left( {GBq} \right)}}{{n_{A} \left( {\mu mol} \right)}} $$

### In vitro human plasma stability

Human plasma, 600 µL, was mixed with formulated tracer solution, 100 µl. The plasma/tracer solution was vortexed to ensure thorough mixing, then incubated at 37 °C. Three time points were selected for HPLC measurements: 0, 15, and 30 min. The 0 min sample was processed immediately after mixing. A fourth control sample (PBS: tracer = 6:1) was also measured at 30 min.

To precipitate the plasma proteins, the solution was mixed with 600 µL ACN and vortexed. The mixture was centrifuged at 16,000 X g for 3–4 min (Eppendorf 5415R centrifuge) at 4 °C, and the supernatant was removed and filtered through a low protein-binding nylon membrane filter (0.2 µm) twice. The filtered solution was stored at 4 °C, and 5–10 µL was subjected to HPLC analysis. PBS/tracer samples were injected after 30 min incubation without further manipulation (HPLC method: AMF (A)/ ACN (B) gradient, solvent B: 10–30%, 6 min, retention time: 4 min) (VWR Hitachi Chromaster, Avantor, Pennsylvania, USA).

### In vitro autoradiography

Binding of [^11^C]Methyl-V-0219 was evaluated through autoradiographic analysis using frozen sections derived from cell pellets expressing human GLP1 receptor selectivity, glucose-dependent insulinotropic polypeptide (GIP), or glucagon (GCG) receptors (huGLP1-R-HEK293, huGIPR-HEK293, huGCG-HEK293), and the rodent insulinoma (INS-1) cell line. Tissue sections were incubated at room temperature for 40 min in PBS containing 1% BSA, either with [^11^C]Methyl-V-0219 at a concentration of 1 MBq/mL (corresponding to approximately 1–4 nM of compound) alone or co-incubated with 20 μM of a competitive blocking agent (Methyl-V-0219 or V-0219). After the incubation period, sections were washed three times (× 1 min) with cold PBS, dipped in Milli-Q water, and then air-dried at 37 °C for 8 min. Subsequently, the samples were exposed to digital phosphor imaging plates (BAS-MS, Fujifilm) for a minimum of four hours, corresponding to more than ten half-lives of the radiotracer. A 10 μL droplet from the radioactive assay buffer was included as a reference for quantitative analysis. The exposed plates were scanned using a phosphor imaging system (Amersham Typhoon FLA 9500, GE Healthcare) at a resolution of 50 μm per pixel. Image analysis of the resulting autoradiograms was conducted using ImageJ software (NIH, USA). Statistical analysis was performed using one-way ANOVA.

### Animal welfare

Animal experiments were conducted in rats and pigs. Male Sprague–Dawley rats (n = 10, body weight 334 ± 45 g) were housed in pairs under controlled environmental conditions (25 °C, 50% relative humidity) on a 12 h light/dark cycle with ad libitum access to food and water.

Two pigs (Yorkshire × Swedish Landrace × Hampshire; body weight 30.5 ± 0.45 kg) were obtained from a commercial breeder and transported to the imaging facility according to established local procedures.

All animal procedures were approved by the Uppsala Animal Ethics Committee and conducted in accordance with Uppsala University guidelines for animal research (UFV 2007/724) and the ARRIVE guidelines.

### [^11^C]Methyl-V-0219 vs [^68^Ga]Ga-DO3A-Exendin-4 direct PET/CT comparison in pig

Two pigs were included in this study, and each animal underwent two PET/CT imaging sessions on the same day. In the morning, an intravenous injection of [^11^C]Methyl-V-0219 (344.9 ± 32.3 MBq) was administered, followed by a 60-min dynamic PET acquisition. During this acquisition, either the brain or the pancreas was positioned within the field of view (FOV). A whole-body PET scan was subsequently performed.

In the afternoon, the same animals received an intravenous injection of [^68^Ga]Ga-DO3A-Exendin-4 (33.4 ± 0.8 MBq) and were imaged using an identical protocol, consisting of a dynamic PET acquisition followed by whole-body imaging.

All animals were anesthetized, ventilated, and continuously monitored according to previously described procedures (Perchiazzi et al. 2014). For imaging, the pigs were positioned supine in the PET/CT scanner. A CT scan was acquired for attenuation correction and anatomical reference using a 64-slice digital 4-ring system with an axial FOV of 198 mm. Acquisition parameters included 100 kV, 80–400 mA, a noise index of 10, a rotation time of 0.5 s, helical acquisition mode, slice thickness of 3.75 mm, a pitch of 0.98:1, and a reconstruction diameter of 50 mm.

Dynamic PET data were acquired in 30 frames with the following temporal sequence: 12 × 10 s, 6 × 30 s, 5 × 2 min, 5 × 5 min, and 2 × 10 min. Arterial blood samples were collected at 5, 30, 60, and 90 min after tracer injection to measure radioactivity concentrations in plasma and whole blood.

PET images were reconstructed using the VPFX-S iterative algorithm (GE Healthcare) with 3 iterations, 16 subsets, a 3-mm Gaussian post-filter, and a matrix size of 256 × 256.

At the end of the imaging protocol, the animals were euthanized under deep anesthesia by intravenous administration of potassium chloride (KCl).

### [^11^C]Methyl-V-0219 vs [^68^Ga]Ga-DO3A-Exendin-4 PET/MRI comparison in rats

Three animals received an intravenous tail vein injection of [^11^C]Methyl-V-0219 (29.5 ± 6 MBq, 16.8 ± 6 µg/kg), while two received [^68^Ga]Ga-DO3A-Exendin-4 (6.8 ± 0.1 MBq, 0.1 ug/kg) and underwent a dynamic 60 min PET scan with the brain in the FOV.

In addition, two rats received [^11^C]Methyl-V-0219 (29.5 ± 6 MBq, 16.8 ± 6 µg/kg) and underwent dynamic 60 min PET scan focused on the abdominal region to assess tracer biodistribution. All procedures were performed under general anesthesia induced and maintained with isoflurane. Imaging was conducted using a nanoPET/MRI system (Mediso Medical Imaging Systems). The dynamic PET imaging was followed by a whole-body PET scan. Subsequently, an MRI was performed for anatomical reference and attenuation correction. After completion of the imaging protocol, all animals were euthanized using CO_2_ gas under anesthesia.

### In vivo blocking and competition studies

An additional group of three rats was included to evaluate tracer specificity. All animals were anesthetized with isoflurane and positioned in the nanoPET/MRI system (Mediso Medical Imaging Systems).

Each rat received an intravenous injection of [^11^C]Methyl-V-0219 (29.5 ± 6 MBq, 0.9 ± 0.4 µg/ml) via the tail vein. Two rats underwent pre-blocking with unlabeled Methyl-V-0219, in which the Methyl-V-0219 (1 mg/kg) was administered 10 min before tracer injection. One rat underwent a competition (displacement) study, where the Methyl-V-0219 (1 mg/kg) was administered 20 min after tracer injection.

Dynamic PET imaging was performed for 60 min post-tracer administration, followed by a whole-body PET scan and a subsequent MRI acquisition. By the end of the study, the animals were euthanized via CO2 under general anesthesia.

## Results

### Radiochemistry

After approximately 30 min of irradiation at 45 µA, cyclotron-produced [^11^C]CO_2_ was transferred to the TPS module where it was converted to [^11^C]CH_3_I. The starting activity of [^11^C]CO_2_ activity was in the range of 50–60 GBq. The [^11^C]CH_3_I was transferred to a vial containing the precursor (**1d**) and the Suzuki–Miyaura reaction mixture. The activity trapped in the vial was defined as the starting activity for evaluation of radiochemical yield, RCY, and amounted to 24.6 ± 6.1 GBq.

The radiolabeling reaction (Scheme 1) was evaluated using two solvent systems: THF/H_2_O (8.75:1) and DMF/H_2_O (8.75:1) (Farhang et al. 2022). The reaction proceeded in both solvent mixtures, and no significant differences in activity yield were observed, although the Pd_2_(dba)_3_/P(o-tol)_3_ catalyst system showed poorer solubility in DMF than in THF. As previously reported, the presence of water in the solvent system can facilitate hydrolysis or activation of boronate ester precursors and improve Suzuki–Miyaura coupling performance thorough suppression of the formation of boroxines in solution (Rokka et al. 2021). In addition, reactions performed in DMF/H_2_O at 70 °C and 80 °C gave comparable results.

RCY after SPE-based reformulation was initially lower, 21 ± 5% (n = 5) due to incomplete recovery from the SPE cartridge. This was improved by a minor adjustment of the ethanol elution volume from 0.5 to 0.7 mL, resulting in an RCY of 43 ± 17% (n = 11) (Supplementary Figure S3). The overall RCY, pre- and post-optimization, was 36 ± 18% (n = 16). After optimization, [^11^C]Methyl-V-0219 was obtained in non-decay-corrected activity yields of up to 4.9 GBq, with a mean of 3.3 ± 1.2 GBq (n = 11), and a formulation ethanol content of 11–14%. Co-injection of the product solution with methyl-V-0219 confirmed the identity of the product as [^11^C]Methyl-V-0219 (Supplementary Figure S4).

### In vitro human plasma stability

In vitro stability assays using radio-HPLC demonstrated that [^11^C]Methyl-V-0219 (A_m_ = 257.5 ± 73.8 GBq/µmol, n = 2) was stable in human plasma with 94.7 ± 0.1% of the parent compound intact after 30 min (Supplementary Fig. 5). A parallel stability assay conducted in PBS formulation buffer showed comparable results with 92.5 ± 1.1% of the radiotracer remaining intact after 30 min.

### In vitro autoradiography

**Fig. 2:**
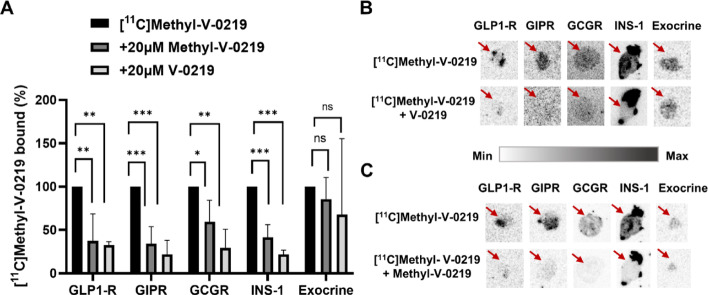
In vitro binding of [^11^C]Methyl-V-0219. **A** Comparison of total binding of [^11^C]Methyl-V-0219 to frozen human cell sections expressing GLP1-R, GIPR, and GCGR, as well as to INS-1 and exocrine tissue sections, following incubation with the radiotracer alone or in the presence of the Methyl-V-0219 or V-0219 blocking agents (one-way ANOVA. *p < 0.05, **p < 0.01, ***p < 0.001, ns = not significant). **B** Representative autoradiograms illustrating reduced uptake of [^11^C] Methyl-V-0219 in various frozen sections, influenced by the blocking effect of V-0219 (see red arrows). **C** Representative autoradiograms demonstrating reduced binding of [^11^C]Methyl-V-0219 in sections pre-incubated with Methyl-V-0219 (see red arrows)

[^11^C]Methyl-V-0219 demonstrated binding to cells expressing both human and rodent GLP-1R (huGLP-1R HEK293 cell sections and INS-1 sections, respectively) (Fig. 2A–C). Pre-incubation with V-0219 reduced [^11^C]Methyl-V-0219 binding to GLP-1R and INS-1 sections by more than 65% (Fig. 2A–B; *p* < 0.001). A similar inhibitory pattern was observed following pre-incubation with Methyl-V-0219. In this case, tracer binding was reduced by over 75% to GLP-1R expressing sections and by 44% to INS-1 sections (Fig. 2A, C; *p* < 0.001).

Somewhat surprisingly, [^11^C]Methyl-V-0219 also exhibited consistent and significant cross-reactivity with GIPR- and GCGR-expressing cell sections. Binding to GIPR- and to a lesser extent GCGR-expressing cells was also consistently reduced by co-incubation with either V-0219 or Methyl-V-0219. Although the degree of binding to GCGR was more variable, it remained clearly blockable.

In contrast, [^11^C]Methyl-V-0219 showed no significant or blockable binding to exocrine pancreatic tissue sections, which contain no endocrine cells and are negative for both GLP-1R, GIPR, and GCGR (Fig. 2A–C).

### [^11^C]Methyl-V-0219 vs [^68^Ga]Ga-DO3A-Exendin-4 direct PET/CT comparison in pig

**Fig. 3:**
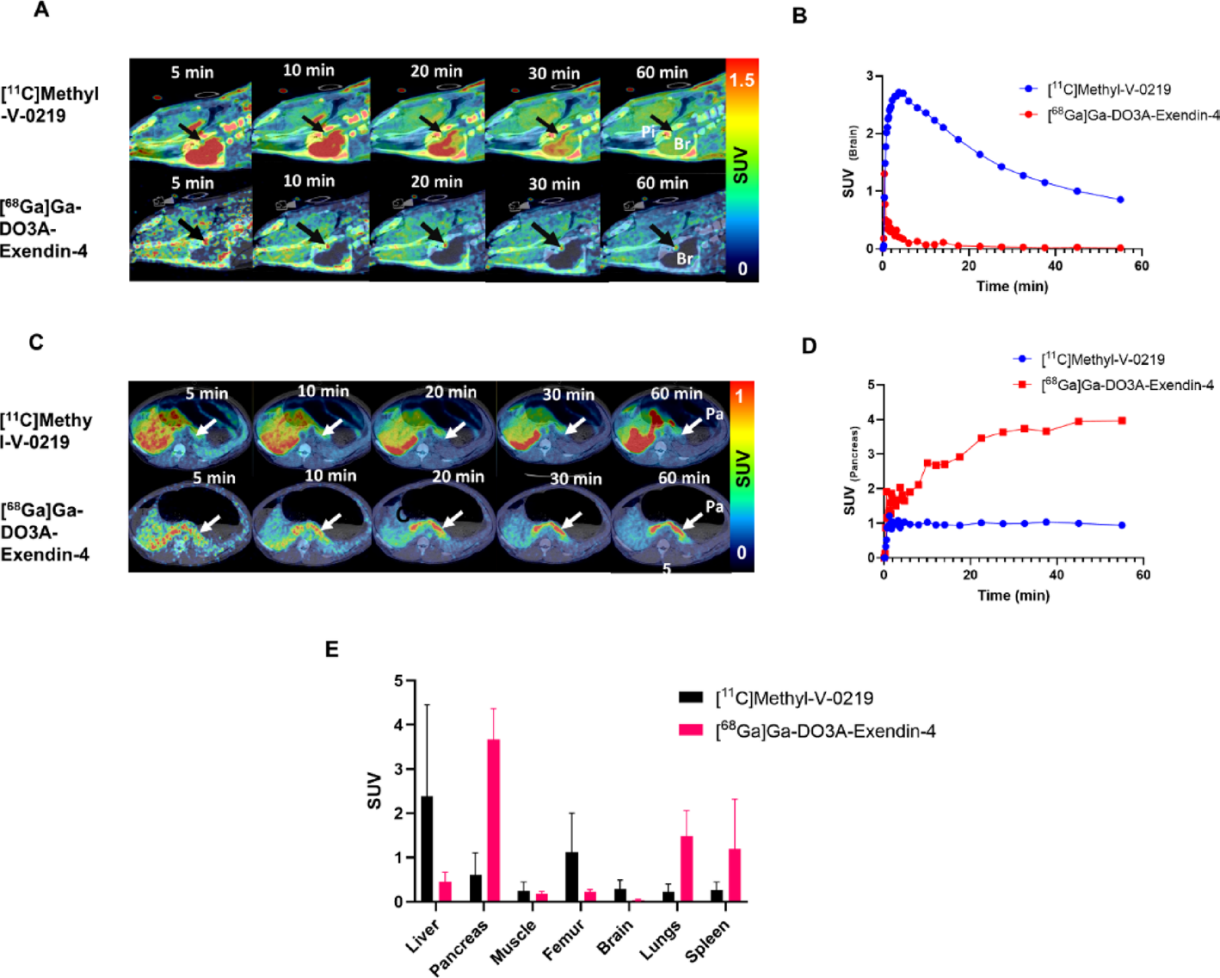
In vivo PET biodistribution of [^11^C]Methyl-V-0219 in pigs. **A** Representative sagittal PET/CT images acquired at multiple time points following intravenous administration of [^11^C]Methyl-V-0219 (upper panel) and [^68^Ga]Ga-DO3A-Exendin-4 (lower panel) during dynamic brain imaging in pig. Br, brain; Pi, pituitary gland. Arrowheads indicate the pituitary gland. **B** Time–activity curves showing brain uptake kinetics of [^11^C]Methyl-V-0219 and [^68^Ga]Ga-DO3A-Exendin-4 during the 60-min dynamic PET acquisition. **C** Representative transverse PET/CT images of the pancreatic region acquired at multiple time points following intravenous administration of [^11^C]Methyl-V-0219 (upper panel) and [^68^Ga]Ga-DO3A-Exendin-4 (lower panel). Pa, pancreas. Arrowheads indicate the pancreas. **D** Time–activity curves showing pancreatic uptake kinetics of [^11^C]Methyl-V-0219 and [^68^Ga]Ga-DO3A-Exendin-4 during the dynamic PET acquisition. **E** Quantitative SUV analysis of selected organs 60 min after tracer administration. Data are presented as SUV mean ± SD of n = 2

Dynamic PET imaging of the brain demonstrated rapid uptake of [^11^C]Methyl-V-0219, with SUV values exceeding 2 within the first 5 min post-injection, followed by a gradual washout to SUV below 1 by the end of the 60-min acquisition (Fig. 3A). In contrast, increased uptake over time was observed in harderian glands, regions surrounding the eyes, and salivary glands, suggesting progressive tracer accumulation in these tissues.

Compared with [^68^Ga]Ga-DO3A-Exendin-4, which showed pronounced retention consistent with GLP-1 receptor binding in the pituitary gland, [^11^C]Methyl-V-0219 did not exhibit noticeable retention in this region (Fig. 3A).

No noticeable uptake of [^11^C]Methyl-V-0219 was observed in the pancreas, whereas [^68^Ga]Ga-DO3A-Exendin-4 demonstrated strong accumulation consistent with GLP-1 receptor expression (Fig. 3C).

In contrast, [^11^C]Methyl-V-0219 exhibited high hepatic retention, followed by increasing activity in the intestines, consistent with hepatobiliary clearance typically observed for lipophilic compounds (Fig. 3E).

### [^11^C]Methyl-V-0219 vs [^68^Ga]Ga-DO3A-Exendin-4 PET/MRI comparison in rats

**Fig. 4:**
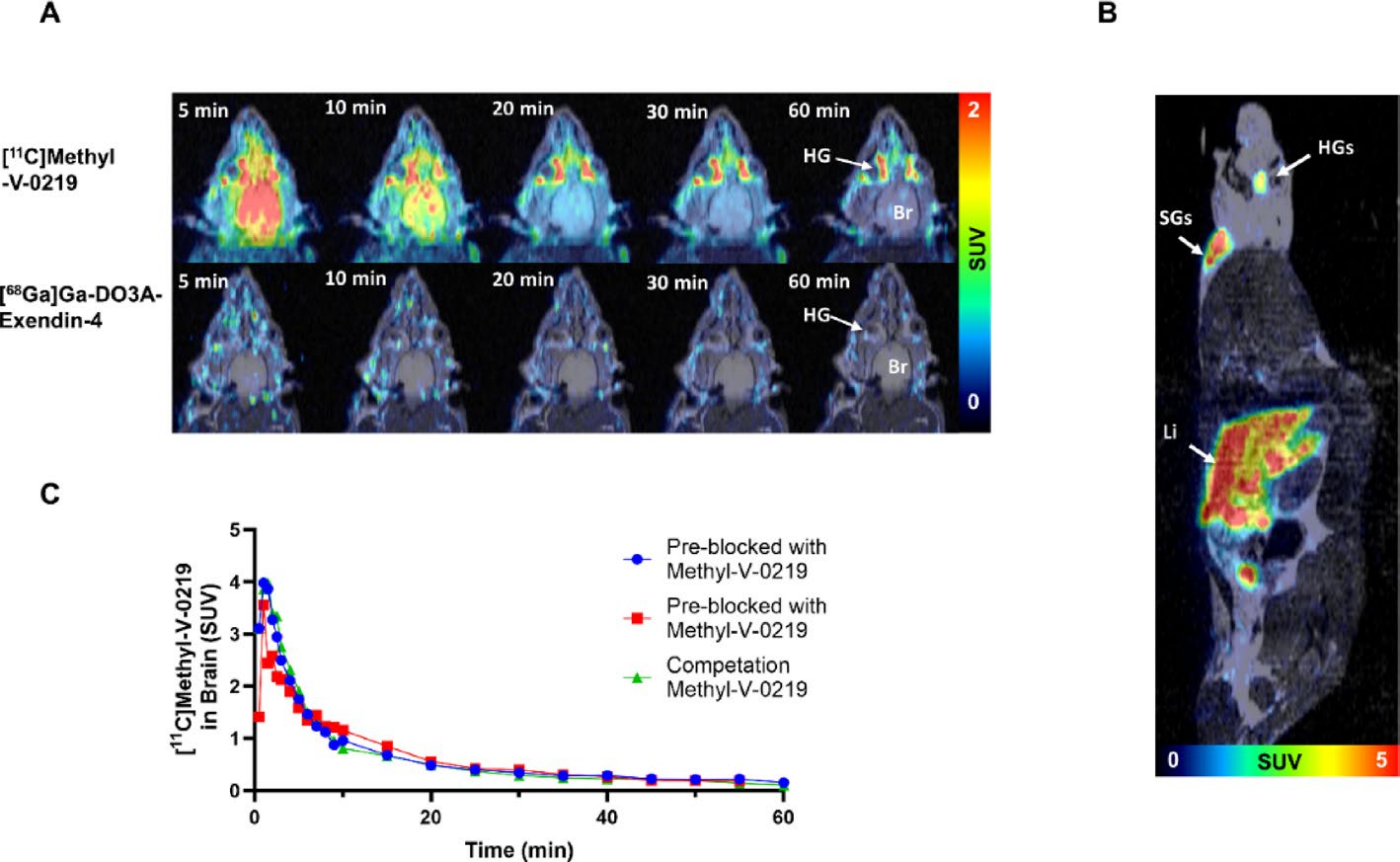
Rat PET imaging and blocking studies. **A** Representative coronal PET/MRI images acquired during dynamic brain scan following intravenous administration of [^11^C]Methyl-V-0219 (upper panel) and [^68^Ga]Ga-DO3A-Exendin-4 (lower panel) in rats at different time points. Arrowheads indicate the harderian glands. Br, brain. HGs, harderian glands. **B** Representative whole-body PET/MRI image acquired 60 min after intravenous administration of [^11^C]Methyl-V-0219. **C** Time–activity curves of [^11^C]Methyl-V-0219 uptake in the rat brain at different blocking conditions. Arrowheads indicate: SGs, salivary glands. Li, liver, and Hgs, harderian glands. SUVs are displayed according to the indicated colour scale

A distribution pattern similar to that observed in pigs was also seen in rats. [^11^C]Methyl-V-0219 exhibited rapid initial uptake in the brain, followed by progressive washout over time (Fig. 4A). No appreciable retention was detected in GLP-1 receptor–rich regions, including the pancreas and pituitary gland. Instead, increasing activity was observed in peripheral tissues, particularly in the liver and intestines, consistent with hepatobiliary clearance (Fig. 4B).

Notably, elevated and progressively increasing uptake of [^11^C]Methyl-V-0219 was also observed in the harderian glands, as well as in salivary glands, suggesting accumulation in glandular tissues (Fig. 4B).

### In vivo blocking and competition studies

Neither pre-blocking nor competition (displacement) with 1 mg/kg of Methyl-V-0219 resulted in any observable change in the brain uptake or kinetic profile of [^11^C]Methyl-V-0219 (Fig. 4C). Time–activity curves demonstrated comparable uptake and washout patterns across all conditions, with no reduction in signal intensity following administration of the blocking agent (Fig. 4C).

Similarly, the competition study, in which the blocking agent was administered 20 min after tracer injection, did not alter tracer retention or distribution in the brain (Fig. 4C).

## Discussion

GLP-1R PET tracers based on the exendin-4 peptide, radiolabeled by coordination of radiometals (primarily gallium-68), to chelators such as DOTA or NODAGA, are currently being clinically evaluated for insulinoma diagnosis (Boss et al. 2024). Additionally, exendin-4 PET probes have shown significant promise for the detection of human transplanted islets (Carlsson et al. 2025) and quantification of dual agonist drug occupancy at the GLP-1R in the pancreas, as well as GLP-1R expressing tumors (Jansen et al. 2023); (Eriksson et al. 2020); (Körner et al. 2007). Interestingly, gallium-68 labeled exendin-4 has also demonstrated strong binding to circumventricular organs (CVOs) close to the brain, such as the pituitary (Eriksson et al. 2022), known to express GLP-1R in high concentrations. Importantly, gallium-68 labeled exendin-4 can even detect drug target engagement at the GLP-1R in CVOs (Khalil et al. 2025). However, exendin-4-based PET tracers are unsuitable for passage across the BBB due to their size, charge, and hydrophilicity, and are thus not optimal for the detection of GLP-1R in the parts of the brain protected by the BBB, such as the appetite center in the hypothalamus.

The most straightforward approach to enable PET imaging of receptors in the brain is to use a scaffold amenable to passage of the BBB, such as small molecules with physicochemical properties compatible with BBB penetration. Thus, herein we present a first-in-class small molecule-based GLP-1R PET tracer, based on the allosteric GLP-1R modulator V-0219.

The Suzuki–Miyaura reaction enabled reliable synthesis of [^11^C]Methyl-V-0219 with high activity yields and high radiochemical purity. The tracer was produced in a ready-for-injection formulation with high molar activity. The synthesis was robust and could be performed using either THF/H_2_O or DMF/H_2_O, with no significant difference in radiochemical yield. The complete synthesis was performed in less than 30 min, which is compatible with the time constraints of carbon-11 chemistry.

The results confirm that both THF/H_2_O and DMF/H_2_O are viable solvent systems for Suzuki–Miyaura ^11^C-methylation of [^11^C]Methyl-V-0219. The presence of water plays a crucial role in maintaining labeling efficiency by promoting ester hydrolysis and minimizing boroxine formation, as previously described (Rokka et al. 2021; Milicevic Sephton et al. 2020; Koyama et al. 2013; Doi et al. 2009; Watanabe et al. 2014; Molander and Canturk 2009; Carrow and Hartwig 2011; Lennox and Lloyd-Jones 2014; Ahmed et al. 2023; Jagodzinska et al. 2009).

Although the final product was radiochemically pure (98.6 ± 1.6%, n = 16), minor unidentified impurities were observed in the UV trace. Suboptimal recovery from the C18 SPE cartridge initially reduced the radiochemical yield, but this was amended by increasing the ethanol elution volume from 0.5 to 0.7 mL.

Overall, the optimized radiosynthesis, including SPE-based reformulation, reproducibly provided [^11^C]Methyl-V-0219 in GBq quantities with > 95% radiochemical purity and high molar activity in a PBS-based formulation suitable for subsequent in vitro characterization and in vivo PET studies.

The in vitro autoradiography assay demonstrated retained binding of [^11^C]Methyl-V-0219 to both human and murine GLP-1R-expressing tissues. Co-incubation with excess unlabeled Methyl-V-0219 markedly reduced radiotracer binding, indicating the presence of a displaceable binding component in GLP-1R-expressing tissues. Similar results were observed in blocking experiments performed with V-0219. Similarly, blocking assays using V-0219 showed comparable outcomes. This is consistent with the differences in bioisoteric relationship between the CH_3_ and CF_3_ moieties (Jagodzinska et al. 2009). The binding assay results also confirm that methyl-V-0219 has a similar binding profile to V-0219.

Surprisingly, [^11^C]Methyl-V-0219 also demonstrated binding to GIP receptor and to a lesser extent GCG receptor expressing tissues, indicating limited receptor selectivity. This has not been previously reported (Decara et al. 2022), but is not entirely unexpected as the GIP and GCG receptors are partially homologous to the GLP-1R. In fact, the endogenous GIP(1–42) ligand can activate the human GLP-1R receptor at high doses, and similarly, endogenous GLP-1 can activate GIPR (Wang 2022). Although V-0219 has previously been shown to exert GLP-1R-dependent pharmacological effects, including loss of activity in GLP-1R knockout mice and blockade by the GLP-1R antagonist exendin (9–39), the original study did not evaluate binding or functional activity at GIPR or GCGR (Decara et al. 2022). Therefore, the receptor binding observed in the present study cannot be directly compared with a previously established selectivity profile.

Prior to in vivo evaluation, [^11^C]Methyl-V-0219 was confirmed to be stable in human plasma and PBS formulation buffer over the relevant experimental time frame. Across both pigs and rats, [^11^C]Methyl-V-0219 exhibited similar biodistribution patterns, characterized by limited retention in target tissues, such as the pancreas and pituitary gland, combined with prominent uptake in the liver and intestines, indicating hepatobiliary clearance (Fig. 3B). However, [^11^C]Methyl-V-0219 readily passed the BBB and exhibited strong uptake in brain rapidly after injection in both rats and pigs. By contrast, [^68^Ga]Ga-DO3A-Exendin-4 did not demonstrate uptake in most brain areas protected by the BBB.

Additionally, increased uptake in glandular tissues, including harderian and salivary glands, may reflect off-target accumulation, driven probably by tracer’s lipophilicity, rather than receptor-mediated binding. Such uptake is commonly observed in PET imagingof lipophilic compounds and has been previously reported in salivary and Harderian glands, where it is attributed to nonspecific binding, passive diffusion, or hepatobiliary-associated excretion mechanisms rather than receptor-mediated retention (Wang 2022); (Kim et al. 2014); (Afshar-Oromieh et al. 2013). The progressive accumulation observed in the regions surrounding the eyes and in salivary tissues in the present study is therefore likely associated with the physicochemical properties of [^11^C]Methyl-V-0219. In particular, the lipophilic nature of the tracer, together with the lipid-rich composition of glandular tissues, may contribute to nonspecific retention in these regions rather than reflecting specific GLP-1R binding.

Importantly, the in vivo blocking and competition studies did not demonstrate any measurable effect on tracer uptake in the brain in rats. Neither pre-blocking nor displacement conditions altered the time–activity curves (Fig. 4C), suggesting that the observed brain signal was not driven by specific, displaceable binding to GLP-1R but instead primarily reflected non-specific uptake and perfusion. This contrasts with the receptor-specific binding observed in the in vitro autoradiography experiments, which were performed using receptor-overexpressing cell models that are not directly comparable to physiological receptor expression in vivo. Although GLP-1R expression in the brain is lower than in the pancreas (Pyke et al. 2014), which could limit the receptor-specific PET signal in the CNS, the lack of appreciable tracer retention in the pancreas despite its relatively high GLP-1R expression indicates that the absence of measurable in vivo specificity cannot be explained solely by low receptor density in the brain. These experiments were performed in a limited number of animals and should therefore be considered exploratory; nevertheless, no evidence of displaceable GLP-1R-specific binding was observed under the experimental conditions evaluated. This apparent discrepancy with the previously reported pharmacological activity of the parent compound may reflect the different requirements for pharmacological efficacy and PET imaging. While V-0219 has been shown to produce GLP-1R-mediated effects, pharmacological efficacy can be achieved at relatively low receptor occupancy, whereas PET imaging requires sufficient receptor-specific binding to generate a measurable signal. Consequently, receptor-specific binding of [^11^C]Methyl-V-0219 may have been below the detection threshold for PET under the experimental conditions used. These findings, combined with the peripheral biodistribution profile and in vitro results, limit the suitability of [^11^C]Methyl-V-0219 as a PET tracer for imaging GLP-1R in vivo.

## Conclusion

[^11^C]Methyl-V-0219 was synthesized using Suzuki–Miyaura ^11^C-methylation, providing the tracer in useful radiochemical yield and high radiochemical purity suitable for biological evaluation. The labelled compound was stable in human plasma and showed blockable binding to GLP-1R in vitro, but also demonstrated binding to GIP and GCG receptors. In vivo studies showed rapid brain uptake consistent with BBB-penetration, but limited retention in GLP-1R–rich tissues and no detectable displaceable binding under the experimental conditions. Together, these findings demonstrate the feasibility of developing small–molecule–based PET tracers targeting incretin receptors, while highlighting the challenges associated with achieving sufficient receptor selectivity and in vivo specificity, particularly for brain imaging applications. These results provide a framework for the rational design of next‑generation small‑molecule PET tracers with improved receptor selectivity and in vivo specificity.

## Supplementary Information

Below is the link to the electronic supplementary material.

Supplementary Material 1

Supplementary Material 2

## Data Availability

The datasets used and/or analysed during the current study are available from the corresponding author on reasonable request.
