# Supplementary material for: Radiosynthesis and preclinical evaluation of [11C]Methyl-V-0219, a small molecule glucagon-like peptide-1 allosteric modulator for PET brain imaging
Source: EJNMMI Radiopharm Chem. 2026 Aug 14;11:58. doi: 10.1186/s41181-026-00486-4 (PMC13481929; doi:10.1186/s41181-026-00486-4)
Supplement: Supplementary file 1 [file 41181_2026_486_MOESM1_ESM.docx]

**Supplementary Material:**

**Radiosynthesis and preclinical evaluation of [¹¹C]Methyl-V-0219, a small molecule Glucagon-Like Peptide-1 allosteric modulator for PET brain imaging**

Francesco Lechi et Al.

**Supplementary Figures**

**Supplementary Scheme 1.** Full synthesis of BPin-V-0219 (**1d**): (a) Et_3_N, 0 °C, chloroacetyl chloride, reflux 4 h, 77%; (b) **2**, DIPEA, ACN, reflux 4 h, 66%; (c) B_2_Pin_2_, dppf, Pd(dppf)Cl_2_, KOAc, dioxane, 90 ⁰C 3 h, 36%;

**Supplementary Figure S1.** Typical radiochromatograms for QC HPLC (A) and prep-HPLC (B).

**Supplementary Figure S2.** UV calibration curve.

**Supplementary Figure S3.** Comparison of average RCY in relationship with the volume of EtOH used to extract the product from the SPE cartridge. Statistical significance was assessed using an unpaired t-test (P<0.05).

**Supplementary figure S4.** QC chromatogram of [^11^C]Methyl-V-0219, spiked with methyl-V-0219 (t_R_ = 5 min). The slight difference in retention time is a result of the physical distance between the radio and UV detectors (Note: the radio-chromatogram shown here presents impurity peaks at 1 min and 2 min; no other instances of these impurities were ever recorded in any of the other syntheses).

**Supplementary Figure S5.** Stability of [^11^C-methyl]V0219 in human plasma and PBS. N=2 repeats per

timepoint.

**Supplementary figure S6.** Copies of NMR Spectra of compound **1b** (Supplementary scheme 1)

**Supplementary figure S7.** Copies of NMR Spectra of compound **1c** (Supplementary scheme 1)

**Supplementary figure S8.** Copies of NMR Spectra of compound **1d** (Supplementary scheme 1)

**Supplementary Tables**

**Table S1.** Development of the QC method.

| Solvent A (%) | Solvent B (%) | T_ret_ (min) |
| --- | --- | --- |
| 50 | 50 | 0.5 |
| 80 | 20 | 2.7 |
| 90 | 10 | n/a |
| 85 | 15 | 6.7 |

| Solvent A _start_ (%) | Solvent B _start_ (%) | Solvent A _end_ (%) | Solvent B _end_ (%) | T_ret_ (min) |
| --- | --- | --- | --- | --- |
| 95 | 5 | 75 | 25 | 8.1 |
| 85 | 15 | 70 | 30 | 4 |
| 90 | 10 | 60 | 40 | 4.6 |
| 95 | 5 | 60 | 40 | 5.6 |

**Table S2.** Development of preparative HPLC separation method. A (*) highlights peaks with a wide and flat shape.

| Solvent A (%) | Solvent B (%) | T_ret_ (min) |
| --- | --- | --- |
| 60 | 40 | 5 |
| 70 | 30 | 8.5-11.5* |
| 65 | 35 | 5.7-7* |

| Solvent A _start_ (%) | Solvent B _start_ (%) | Solvent A _end_ (%) | Solvent B _end_ (%) | T_ret_ (min) |
| --- | --- | --- | --- | --- |
| 70 | 30 | 40 | 60 | 7.5 |
| 70 | 30 | 30 | 70 | 7.25 |
