# Supplementary material for: Radiosynthesis and preclinical evaluation of [11C]Methyl-V-0219, a small molecule glucagon-like peptide-1 allosteric modulator for PET brain imaging
Source: EJNMMI Radiopharm Chem. 2026 Aug 14;11:58. doi: 10.1186/s41181-026-00486-4 (PMC13481929; doi:10.1186/s41181-026-00486-4)
Supplement: Supplementary file 2 [file 41181_2026_486_MOESM2_ESM.docx]

**Radiosynthesis and preclinical evaluation of [¹¹C]Methyl-V-0219, a small molecule Glucagon-Like Peptide-1 allosteric modulator for PET brain imaging**

Francesco Lechi^1*^, Amina Khalil^1*^, Pablo Novales Bautista^1^, Jakob Öberg^2^, Lorenzo J.I. Balestri^2^, Olof Eriksson^1§^, Jonas Eriksson^1,3^, Luke R. Odell^2^

**Supplementary**

**Methods**

***Precursor and reference chemistry***

Analytical thin-layer chromatography (TLC) was performed on silica gel 60 F-254 plates visualized with UV light. Flash column chromatography was performed using silica gel 60 (40–63 μm). ^1^H NMR spectra were recorded at 400 MHz and ^13^C{^1^H} NMR spectra were recorded at 101 MHz. The chemical shifts for ^1^H NMR and ^13^C{^1^H} NMR are referenced to TMS via residual solvent signals (^1^H: CDCl_3_ at 7.26 ppm, ^13^C{^1^H}: CDCl_3_ at 77.16 ppm). High-resolution molecular masses (HRMS) were determined on a mass spectrometer equipped with an ESI source and a time-of-flight (TOF) mass analyzer or a matrix-assisted laser-desorption ionization source with a Fourier transform ion cyclotron resonance mass analyzer, as indicated. All chemicals were purchased from commercial sources. Compounds **1a**-**1d** and **2** were synthesized according to literature procedures [8] (Supplementary Scheme 1).

***Synthesis of 5-Chloromethyl-3-(4-(bromo)phenyl)-1,2,4-oxadiazole***

To a solution of 4-bromobenzamidoxime (181 mg, 0.84 mmol) in toluene (6 mL) was added Et_3_N (114 µL, 0.82 mmol). The mixture was cooled to 0 °C, and a solution of chloroacetyl chloride (65.2 µL, 0.82 mmol) in 1.8 mL of toluene was added dropwise, followed by an additional 2.5 mL of toluene. The mixture was then heated at reflux for 4 h, cooled to room temperature, and evaporated under reduced pressure. The residue was taken up in EtOAc (30 mL) and washed with distilled water (2 x 20 mL) and brine (20 mL). The organic layer was separated, dried over MgSO_4_, filtered, and concentrated in vacuo. The resulting residue was purified by flash chromatography (1:1 EtOAc/hexane) to afford the title compound **1b** (Scheme 1) as a pale brown solid (173 mg, 77% yield). **Rf** = 0.86 (EtOAc/hexane 1:1); ^1^H NMR (400 MHz, CDCl_3_) δ 7.96 (d, *J =* 8.0 Hz, 2H), 7.64 (d, *J =* 8.0 Hz, 2H), 4.75 (s, 2H); ^13^C NMR (101 MHz, CDCl_3_) δ 175.0, 168.7, 132.7, 129.5, 126.7, 125.6, 33.8 (Supplementary Figure S6).

***Synthesis of 4-[[1-[[3-[4-bromophenyl]-1,2,4-oxadiazol-5-yl]methyl]-3-piperidinyl]methyl]morpholine***

To a solution of oxadiazole **1b** (52 mg, 0.19 mmol) in MeCN (2 mL) was added 4-(3-piperidinylmethyl)morpholine (**2**, 38 mg, 0.21 mmol) and DIPEA (210 µL, 1.20 mmol). The reaction mixture was stirred at reflux for 4 h, cooled to room temperature, and concentrated under reduced pressure. The residue was taken up in EtOAc (30 mL) and washed with distilled water (2 x 20 mL) and brine (20 mL). The organic layer was separated, dried over MgSO_4_, filtered, and concentrated in vacuo. The resulting residue was purified by flash chromatography (99:1 DCM/MeOH) to afford the title compound as a yellow oil (54 mg, 66% yield). **Rf** = 0.72 (DCM/MeOH 99:01); MS (ESI) calculated for C_19_H_26_BrN_4_O [M+H]^+^: 421.1, 423.1 (1:1); Found 421.3, 423.3 (1:1); ^1^H NMR (400 MHz, CDCl_3_) δ 7.97 (d, *J* = 8.0 Hz, 2H), 7.62 (d, *J* = 8.0 Hz, 2H), 3.94-3.83 (m, 2H), 3.59-3.73 (m, 4H), 3.03-3.12 (m, 1H), 2.88-2.95 (m, 1H), 2.26-2.48 (m, 4H), 2.08-2.25 (m, 3H), 1.86-1.96 (m, 2H), 1.58-1.79 (m, 3H), 0.83-0.97 (m, 1H); ^13^C NMR (101 MHz, CDCl_3_) δ 177.1, 167.7, 132.3, 129.1, 125.9, 125.8, 67.2, 63.2, 58.7, 54.3, 53.8, 33.4, 29.0, 25.2. (Supplementary Figure S7).

***Synthesis of 4-[[1-[[3-[4-[4,4,5,5-tetramethyl-1,3,2-dioxaborolan-2-yl]phenyl]-1,2,4-oxadiazol-5-yl]methyl]-3-piperidinyl]methyl]morpholine***

A two-neck flask was charged with B_2_Pin_2_ (265 mg, 1.04 mmol), dppf (15 mg, 0.026 mmol), Pd(dppf)Cl_2_ (38 mg, 0.052 mmol), and KOAc (154 mg, 1.56 mmol). The flask was flushed with nitrogen and a solution of **1c** (220 mg, 0.52 mmol) in dioxane, 3 mL, was added. The mixture was heated at 90 °C for 3 h, cooled to room temperature, and concentrated under reduced pressure. The residue was taken up in EtOAc (30 mL) and washed with distilled water (3 x 20 mL) and brine (20 mL). The organic layer was separated, dried over MgSO_4_, filtered, and concentrated in vacuo. The resulting residue was dissolved in 1 mL of DCM, and *n*-pentane was added until a precipitate started forming. The suspension was stirred for 30 minutes at room temperature, decanted, and concentrated in vacuo. This residue was finally purified by flash chromatography (20 to 100% acetone in DCM) to afford the title compound **1d** (Supplementary Scheme 1) as a pale-yellow oil (88 mg, 36% yield). **Rf** = 0.50 (DCM/Acetone 1:1); MS (ESI) calculated for C_25_H_38_BN_4_O_4_ [M+H]^+^: 469.3; Found 469.5; ^1^H NMR (400 MHz, CDCl_3_) δ 8.09 (d, *J* = 8.0 Hz, 2H), 7.91 (d, *J* = 8.0 Hz, 2H), 3.91 (d, *J* = 6.7 Hz, 2H), 3.61-3.72 (m, 4H), 3.09 (d, *J* = 7.0 Hz, 1H), 2.92 (d, *J* = 11.0 Hz, 1H), 2.28-2.49 (m, 4H), 2.10-2.25 (m, 3H), 1.87-1.96 (m, 2H), 1.59-1.78 (m, 3H), 1.36 (s, 12H), 0.85-0.97 (m, 1H); ^13^C NMR (101 MHz, CDCl_3_) δ 176.9, 168.4, 135.3, 129.1, 126.7, 84.3, 67.1, 63.2, 58.7, 54.3, 54.2, 53.8, 33.4, 29.0, 25.1, 25.0 (Supplementary Figure S8).

***Purification and formulation***

[^11^C]Methyl-V-0219 was purified by preparative HPLC (Agilent 1260 Infinity II, Agilent Technologies, California, USA), injecting the reaction mixture onto a Gemini C18 column (250 x 10 mm, 5µm, 110Å; Phenomenex, CA, USA) and employing a gradient mobile phase consisting of ammonium formate (AMF, 50 mM, pH 3.5) and acetonitrile (can, ≥99.9%, HPLC grade), increasing from 30% to 70% ACN over 15 min (retention time approximately 7.5 min). The product-containing fraction was collected, and passed over a Sep-Pak plus light tC18 cartridge (Waters, Massachusetts, USA). [^11^C]Methyl-V-0219 was trapped on cartridge, washed with sterile water (5 mL), and then eluted in ethanol (absolute, 99.9%) followed by phosphate-buffered saline (PBS, 7 mL) to a crimp-sealed 10 mL glass vial. The resulting formulation contained [^11^C]Methyl-V-0219 in a 5-6 mL solution of PBS with approximately 10% (v/v) ethanol. The radiochemical purity (RCP) was assessed via radio-HPLC(Agilent 1290 Infinity II system, Agilent Technologies, California, USA) using a Chromolith Performance RP-18e (100 x 4.6 mm, Merck, Germany). The QC method used a mobile phase consisting of AMF (50 mM, pH 3.5) (A) and ACN (99.9%, HPLC grade) (B) on a gradient (A:B = 90:10 to A:B = 60:40) over 10 min (retention time = 5 min).

**Results**

***Development of quality control method***

The QC analysis method was developed on an Agilent 1290 Infinity II system (Agilent Technologies, California, USA) HPLC system paired with a Chromolith Performance RP-18e (100x4.6 mm) column. The mobile phase composed of aqueous solution of AMF (50 mM, pH 3.5) (A) and ACN (HPLC grade, 99.9%) (B).

The HPLC QC method was established by testing different mobile phase solvent ratios in both isocratic and gradient conditions. A minimal amount of *methyl*-V-0219 reference compound was dissolved in ACN, injected into the Agilent 1290 Infinity II system and the retention time of the reference compound UV peaks were noted (Supplementary Table 1).

The method ultimately selected was: gradient, solvent B 10% to 40%, as it provided a good balance between analyte retention time and peak shape (Supplementary Figure S1A).

***Development of preparative HPLC separation method***

The preparative HPLC separation method was developed on an Agilent 1260 Infinity II HPLC paired with a Gemini C18, 5µm, 110Å (Phenomenex, California USA) column. As previously established, the UV peak signals of the reference compound *methyl*-V-0219 were observed and their retention time and shape were used to choose the ideal separation conditions.

The mobile phase composition was composed of an aqueous solution of HCO_2_NH_4_ (50 mM) (A) and acetonitrile (HPLC grade, 99.9%) (B), same as for the QC method (Supplementary Table **2**).

A minimal amount of *methyl*-V-0219 reference was dissolved in ACN and injected onto the Agilent 1260 Infinity II HPLC system. The method run time was 15 min: an ideal method in this context would have the compound of interest being retained until roughly half of the run time, and have its peak be relatively sharp in order to be more effectively collected.

The method ultimately selected was: gradient, Solvent B 30% to 70% (Supplementary Supplementary Figure S1B).

The preparative HPLC separation method selected provided excellent baseline separation between the desired product and the non-polar impurities observed at 9 and 11.6 min.

RCP was consistently >95% (Supplementary Figure S4), further confirming the robustness of the preparative method and confirming that the following reformulation step did not affect purity.

***Quantification of [^11^C]Methyl-V-0219***

Concentration in the final solution and molar activity was calculated via simple linear regression using the integrated UV signal of the product (Supplementary Figure S2), as per the following equation: Y = 4.31*X – 0.27, where is the integrated area under curve of the UV peak signal of the product and X is the concentration of the product solution. Calculated concentration of the final solution was 0.8±0.4 µg/mL, while the molar activity was 187.3±101.2 GBq/µmol. From a starting 2.18±0.19 µmol of precursor BPin-V-0219, 13.23±5.53 nmol of [^11^C]Methyl-V-0219 were isolated in the final formulation.
